# Computational AI Models for Investigating the Radiation Shielding Potential of High-Density Concrete

**DOI:** 10.3390/ma15134573

**Published:** 2022-06-29

**Authors:** Muhammad Nasir Amin, Izaz Ahmad, Mudassir Iqbal, Asim Abbas, Kaffayatullah Khan, Muhammad Iftikhar Faraz, Anas Abdulalim Alabdullah, Shahid Ullah

**Affiliations:** 1 Department of Civil and Environmental Engineering, College of Engineering, King Faisal University, Al-Ahsa 31982, Saudi Arabia; kkhan@kfu.edu.sa (K.K.); 218038024@student.kfu.edu.sa (A.A.A.); 2 Department of Civil Engineering, University of Engineering and Technology Peshawar, Peshawar 25120, Pakistan; izazahmad@uetpeshawar.edu.pk (I.A.); mudassiriqbal29@sjtu.edu.cn (M.I.); asimabbas@uetpeshawar.edu.pk (A.A.); shahid.ullah@uetpeshawar.edu.pk (S.U.); 3 Shanghai Key Laboratory for Digital Maintenance of Buildings and Infrastructure, State Key Laboratory of Ocean Engineering, School of Naval Architecture, Ocean & Civil Engineering, Shanghai Jiao Tong University, Shanghai 200240, China; 4 Department of Mechanical Engineering, College of Engineering, King Faisal University, Al-Ahsa 31982, Saudi Arabia; mfaraz@kfu.edu.sa

**Keywords:** concrete, water-cement ratio, radiation shielding, compressive strength, artificial neural network, gene expression programming

## Abstract

Concrete is an economical and efficient material for attenuating radiation. The potential of concrete in attenuating radiation is attributed to its density, which in turn depends on the mix design of concrete. This paper presents the findings of a study conducted to evaluate the radiation attenuation with varying water-cement ratio (*w*/*c*), thickness, density, and compressive strength of concrete. Three different types of concrete, i.e., normal concrete, barite, and magnetite containing concrete, were prepared to investigate this study. The radiation attenuation was calculated by studying the dose absorbed by the concrete and the linear attenuation coefficient. Additionally, artificial neural network (ANN) and gene expression programming (GEP) models were developed for predicting the radiation shielding capacity of concrete. A correlation coefficient (R), mean absolute error (MAE), and root mean square error (RMSE) were calculated as 0.999, 1.474 mGy, 2.154 mGy and 0.994, 5.07 mGy, 5.772 mGy for the training and validation sets of the ANN model, respectively. Similarly, for the GEP model, these values were recorded as 0.981, 13.17 mGy, and 20.20 mGy for the training set, whereas the validation data yielded R = 0.985, MAE = 12.2 mGy, and RMSE = 14.96 mGy. The statistical evaluation reflects that the developed models manifested close agreement between experimental and predicted results. In comparison, the ANN model surpassed the accuracy of the GEP models, yielding the highest R and the lowest MAE and RMSE. The parametric and sensitivity analysis revealed the thickness and density of concrete as the most influential parameters in contributing towards radiation shielding. The mathematical equation derived from the GEP models signifies its importance such that the equation can be easily used for future prediction of radiation shielding of high-density concrete.

## 1. Introduction

The harnessing of energy released during nuclear reactions can be rightly called an important milestone in technological development. Over time, nuclear technology has found its way into several fields; medical science is one of them. Nuclear technology entails the emission of radionuclides such as gamma rays, X-rays, and neutrons. These radionuclides pose a serious risk to all living things, particularly human beings. Nuclear radiation has the potential to destroy living cells, the building blocks of human beings, hence warranting shielding against their destructive action [1,2]. Protecting humans, structures, and equipment from the harmful effects of radiation is an important concern in nuclear engineering. Due to the increasing use of nuclear technology for a wide range of applications such as treatment of cancer disease, thermal energy production, imaging nuclear fuel, etc. [3,4,5,6] and the associated health hazards that this technology entails, the evaluation of a construction material against nuclear radiation is vital [7].

The material selection for radiation shielding is mainly dependent on the emissions source, type, and the material’s weight [8]. A number of materials has been explored for shielding of nuclear radiation such as iron, lead, polyethylene, graphite, and concrete [9,10,11,12,13,14,15,16,17]. Concrete has been widely used in the construction of nuclear facilities primarily due to its versatility, structural strength, and ability to shield nuclear radiation [18]. Concrete is strong, having a reasonable shielding capacity, and, more importantly, economically viable material for the construction of radiation shielding structures in nuclear power plants, healthcare facilities involving radiology and particle accelerators, etc. The radiation shielding performance of concrete is closely associated with its density, with a direct proportionality between density and shielding potential [19,20]. Several studies have concluded that heavyweight and dense concrete has the ability to significantly improve radiation shielding performance and have several other inherent advantages over other materials [7,8,9,19,20,21,22]. Heavyweight concrete is characterized by its density, which is greater than 2600 kg/m^3^ [23] as compared to ordinary concrete composed of normal weight aggregate having density ranges from 2200 kg/m^3^ to 2600 kg/m^3^ [24]. Density has a profound impact on the structural elements, allowing an appreciable reduction in the thicknesses of protective members while not compromising on shielding performance [25,26,27,28,29,30,31,32]. A high density of concrete is usually attained by using high-density aggregates, such as barite, goethite, magnetite, hematite, serpentine, lead, heavy metal oxide, steel slag, steel shot, and colemanite [33,34,35,36,37,38].

Numerous studies in the past have been conducted to investigate the performance of heavyweight concrete (HWC) based on industry requirements and applications. Most of these investigations, however, have focused on exploring the mechanical and shielding performance of HWC based on the type of aggregate used [12,15,25,39,40]. Izaz et al. [8] conducted a detailed study to evaluate both the mechanical and gamma radiation shielding performance of concrete mixtures produced with barite aggregates. The authors noted that an increase in barite quantity in the concrete mix resulted in an acceptable reduction in strength, a decrease in shrinkage, and a substantial increase in linear attenuation coefficient. Coskun et al. [41] also concluded that barite concrete mixtures successfully achieved the target radiation shielding. Alwaeli and Nadziakiewicz [40] used water iron products such as scale and steel chips as replacement of fine aggregate in proportions of 25, 50, 75, and 100% to evaluate compressive strength and shielding against gamma radiation. They concluded that concrete prepared with steel chips increased the compressive strength as compared to conventional concrete. It was also reported that steel chips also improved the absorption of gamma radiation. In their study, Gencel et al. [15] investigated the mechanical strength and neutron & gamma radiation shielding performance of concrete containing hematite as coarse aggregate. It was revealed that gamma radiation performance and compressive strength of concrete increased with the addition of hematite; however, it did not affect neutron radiation shielding performance. The radiation shielding performance of concrete infused with silica fume and lead powder was explored in a study conducted by Ochbelagh et al. [17] Study results showed that although the addition of silica fume improved the concrete’s compressive strength, it reduced the gamma-ray radiation shielding performance. Azeez et al. [31] conducted an experimental study to evaluate the radiation shielding behavior of heavyweight concrete produced with high-density coarse aggregates such as steel shots, steel slag, and iron ore. Study results showed that radiation performance is affected by the unit weight of HWC mixtures, irrespective of the type of aggregates used. Al-Humaiqani et al. [42] studied the ability of high strength concrete prepared with different types of aggregates against radiation. Laboratory prepared samples were subjected to Cs137 radiation having an energy of 0.663 MeV using NaI scintillation detector. It was observed that the relationship between linear attenuation coefficients and the density of high-performance concrete is linear. Shams et al. [43] studied both hematite and barite in separate and in mixed form. The results showed that both forms improved the linear attenuation coefficient. Akkurt et al. [44] studied the radiation shielding performance of concrete containing zeolite in different proportions, i.e., 0%, 10%, 30%, and 50%. It was concluded that increasing zeolite concentration reduces the linear attenuation coefficient and is not recommended as a first-choice alternative against radiation shielding. Despite resulting in improvement in radiation shielding, most of the materials have an adverse effect on the mechanical properties of concrete, such as a resulting reduction in compressive and tensile strengths, accompanied by the reduced elastic modulus and higher weight loss at elevated temperatures [8].

Water-cement (*w*/*c*) ratio is also an important factor contributing to the strength and radiation shielding properties of heavy weight concrete. Lotfi et al. [45] found that increasing the *w*/*c* ratio significantly reduces the compressive strength of heavy weight concrete, while the mechanical and radiation shielding behavior of concrete has been found to improve with decreasing *w*/*c* ratio. Yang et al. [46] reported that decreasing the water/cement ratio results in improvement of density, compressive strength, and modulus of elasticity in magnetite concrete.

It is evident from the previous studies that the radiation shielding of concrete is a very complex phenomenon which depends on different variables such as material type, density, thickness of barrier, distance, intensity of radiation, and water cement ratio; it is very difficult to identify the most influencing parameters. Moreover, there is no such model available that co-relate all these variables with radiation shielding ability. To overcome the limitation, the machine learning approach can be used to develop a prediction model that co-relate radiation shielding with all the influencing variables using an experimental dataset. In civil engineering, AI technique is one of the most effective tools in machine learning over the past decades to develop predication models that deal with highly nonlinear problems. Previous studies reveal that artificial neural network (ANN) is an effective tool for assessing the concrete performance while considering the effect of multiple parameters such as composition of ingredients, water cement ratio, and quantity of additives [47,48,49,50,51]. Recently, studies have utilized artificial intelligence (AI) and machine learning (ML) methods for the assessment and prediction of radiation shielding performance of concrete mixtures [52,53]. Yadollahi et al. [54] adopted an ANN to predict optimal mixture combinations such as quantity of cement and different additives against radiation shielding. Juncai et al. [55] adopted the least square support vector machine (LS-SVM) to predict the strength of radiation shielding concrete.

In this study, the ANN method was used to develop a model by using our own experimental data that evaluates the concrete mixes’ performance against radiation without compromising the mechanical properties of concrete. High radiation shielding ability could be achieved with the materials having relatively high density [56]. In the design of radiation shielding, thickness of the barrier or wall is an important parameter to be determined that attenuates the radiation to recommended values that depends on a material’s properties. In the present study, high density materials such as magnetite, barite, and hematite were used to increase the attenuation coefficient of concrete. The main objective of the study is to investigate the radiation shielding parameters as well as the concrete materials’ properties to predict a model to obtain optimum values. In the application of the ANN method, *w*/*c* ratio, cement quantity, and slump value were selected as the control factors and compressive strength and linear attenuation coefficient were considered as the quality responses. At the end, optimal values of mixture with consideration of multiple quality characteristics are obtained and verified.

## 2. Experimental Program

### 2.1. Materials

**Figure 1:**
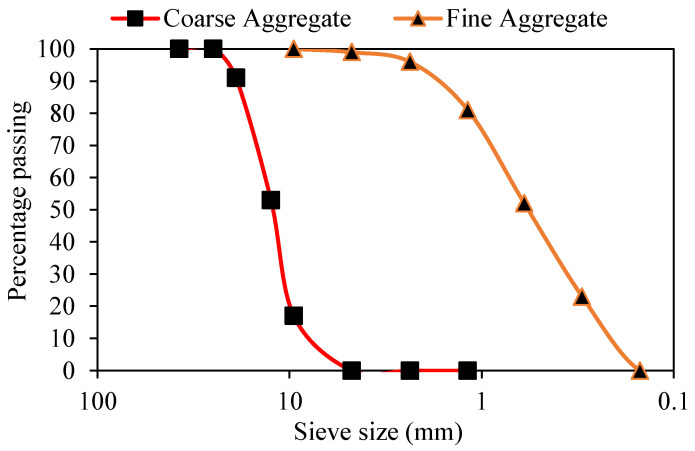
Grading curves of aggregates used in the experimental program.

**Figure 2:**
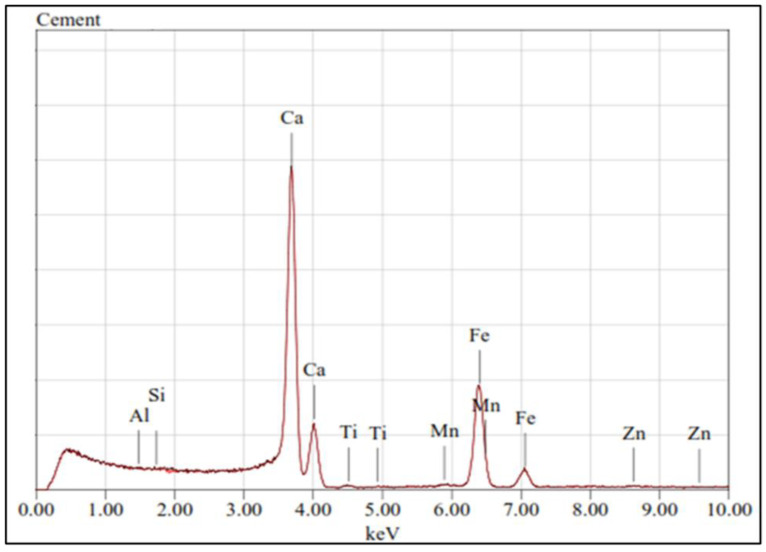
XRF test result of ordinary Portland cement (OPC).

Crushed stone, calcareous in nature (abundant in CaCO_3_), conforming to ASTM C-33, was used as coarse aggregate in concrete, whose particle sizes ranged from 9.5 mm to 25 mm. The gradation curve of the coarse aggregate obtained from sieve analysis (ASTM C136) is presented in Figure 1, which shows the well-graded nature of the coarse aggregates. Naturally available sand, mainly consisting of quartz, was used as fine aggregate. The gradation curve of the sand obtained from sieve analysis (ASTM C33) is presented in Figure 1. The sand was cleaned of any organic impurities by washing it. The gradation curve of sand used in this study was bound by the standard upper and lower limits, as defined by the ASTM standard. Fine aggregate in barite concrete was completely replaced with barite minerals, while it was replaced with magnetite aggregate in magnetite concrete specimens. Ordinary Portland cement conforming to (ASTM C150) was used as a binder in concrete. The characterization of the materials used in this study is presented in Table 1. X-ray diffraction (XRD) test and X-ray fluorescence (XRF) were conducted on cement samples to evaluate their chemical composition. The results of these two tests are presented in Table 2 and Figure 2, respectively.

### 2.2. Mix Proportioning

**Figure 3:**
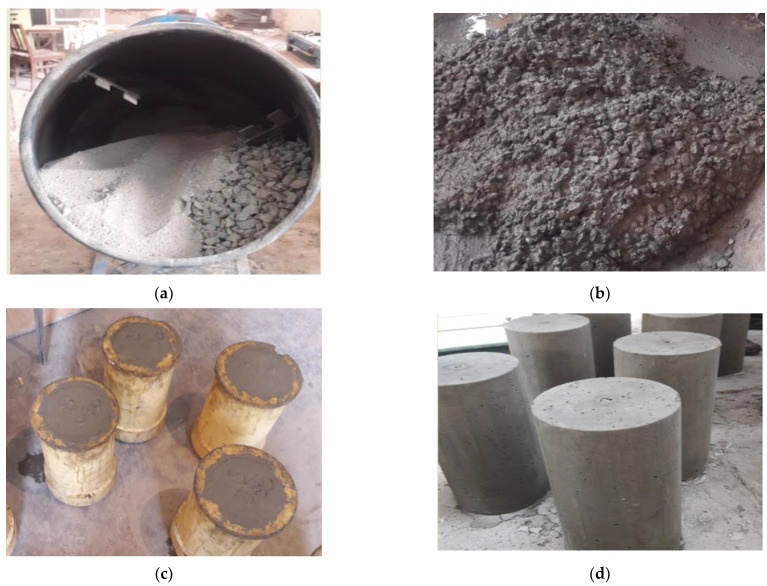
(**a**) concrete mixing, (**b**) fresh concrete, (**c**) sample casting, and (**d**) prepared samples.

Based on the material characteristics, a mix design was carried out for four different *w*/*c* ratios varying between 0.30 and 0.45, with increments of 0.05. For designing the concrete mixes, the required properties of the materials were determined using laboratory tests. The details of mix design are given in Table 3 and Table 4. Ultra-superplasticizer 470 was used as a chemical admixture to increase the workability of concrete. The chemical admixture was added by the weight of the binder. According to ASTM specification, concrete was produced in the laboratory and then cast in cylinders confirming ASTM C470 as shown in Figure 3. Fresh concrete properties are listed in Table 5. Mix design was carried out for normal weight concrete and then the fine aggregate was replaced with barite and magnetite by volume in barite and magnetite added to concrete. The admixture dose was adjusted accordingly to get the targeted slump value.

### 2.3. Testing Program

**Figure 4:**
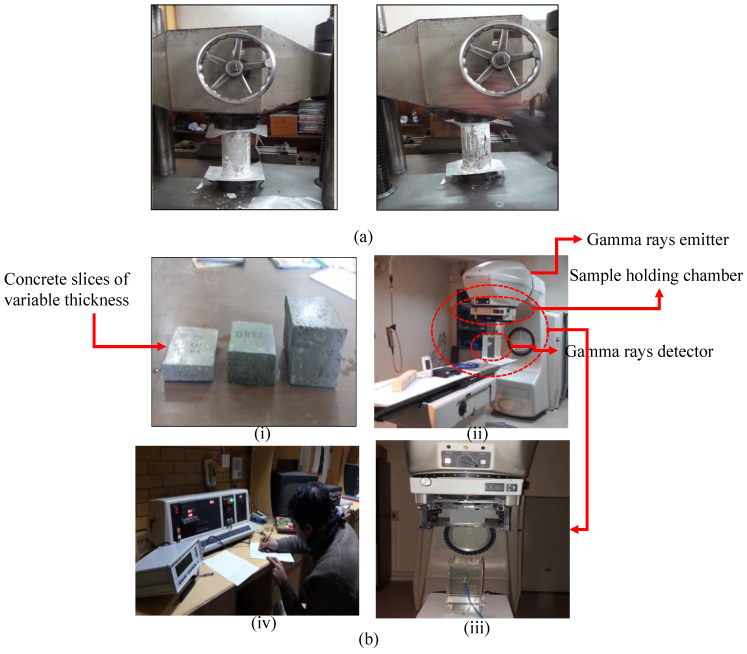
(**a**) test setup for compressive strength, (**b**) Gamma rays’ dosimetry, (**i**) concrete samples for gamma rays’ dosimetry, (**ii**) phoenix machine used as gamma rays’ ejector, (**iii**) zoom in of sample holder and detector, (**iv**) digital data collection.

ASTM C-39 test method was used to determine the compressive strength of concrete. Concrete cylinders of standard dimensions, i.e., 150 mm in diameter and 300 mm in length, were cast using different *w*/*c* ratios. These samples were subjected to a compression test in a universal testing machine. A total of thirty-six concrete cylinders were cast, with three samples representing each *w*/*c* ratio. In this test, concrete cylinders were subjected to axial compressive force until concrete failure. The experimental setup used for compression testing is presented in Figure 4a.

The linear attenuation coefficient was measured to compute the shielding potential of concrete samples. The Phoenix machine, used in cancer treatment for dosimetry, was used to conduct the test. Samples of different thicknesses varying from 2 cm to 10 cm were made with the four *w*/*c* ratios, shown in Figure 4b. Samples were placed in the machine, and the intensity of gamma rays was measured both in the presence and absence of concrete samples. The experimental setup is shown in Figure 4b(i–iv). Then, the correlation given in Equation (1) was used to determine the linear attenuation coefficient:

(1) $$µ=\frac{1}{x}ln\frac{N_{o}}{N}\;$$

where μ = linear attenuation coefficient, *x* = material thickness in cm, *N_o_* = intensity of gamma rays received by the detector in the absence of concrete samples, and *N* = intensity of gamma rays received by the detector in the presence of concrete samples.

### 2.4. Artificial Neural Network Modelling

Artificial neural networks (ANNs) are mathematical based models working on the analogy of the human brain. ANN was started with the concept of the brain which solves computational problems in different ways as compared to conventional computers. A neural network encompasses three layers of immensely equivalent distributed processors such as input, hidden, and output layer. There are multiple factors that impact the neuron quantity in each layer. The number of neurons in the output and input layer is determined through modeling the relevant parameters in the layers. The number of neurons present in the hidden layer is a variable that must be chosen to achieve a suitable output response [57]. In order to train the model, the input and output parametric data are provided to the artificial neuron network. The differences between the predicated and target outputs are adjusted by changing the preferences and weights of the network in order to attain least error [58]. The optimized ANN was obtained by varying the number of neurons in the hidden layer. The Lavenberg–Marquardt function was used for optimizing the weights and preferences of the network, because it is recommended as the top priority function in the case of training supervised algorithms that provide fastest back propagation process [59]. In the output and hidden layers of the network, Purelin and tan-sigmoid were used as activation functions, respectively. In the case of the hidden layer, tan-sigmoid and log-sigmoid functions were used to obtain the best activation function. The tan-sigmoid function provided the optimum outcomes. Based on the previous literature [60,61,62,63,64], correlation coefficient (R), mean absolute error (MAE), and root mean square error (RMSE) was used for statistical evaluation of the ANN model.

For the development of ANN models, the data presented in Table 6 and Table 7, obtained from the experimental data, was employed. It can be seen that three types of concrete, Type 1 (Normal concrete), Type 2 (Barite containing concrete), and Type 3 (magnetite containing concrete), were used. Barite and magnetite were used as replacement of fine aggregate in order to increase the density which can increase the radiation shielding capacity of concrete.

### 2.5. Gene Expression Programming Modelling

**Figure 5:**
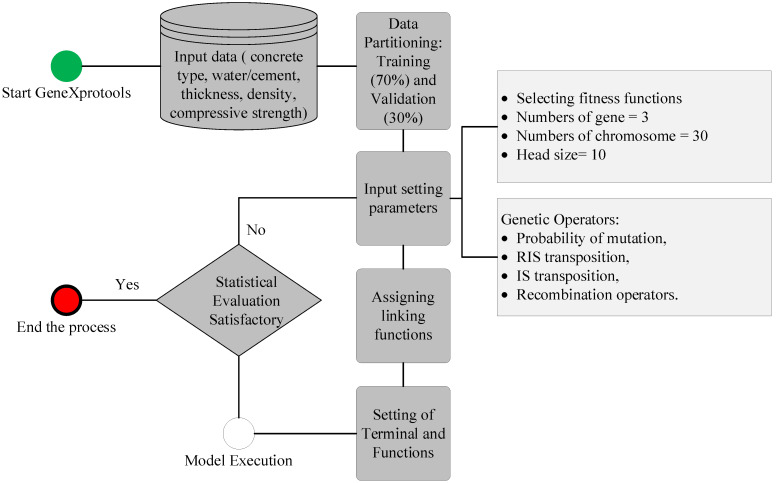
Schematics of gene expression programming (GEP) modelling.

The input data shown in Table 6 and Table 7 was subjected to GeneXprotools for training and validation of the models. The purpose of using the gene expression programming (GEP) model was to develop a mathematical relationship for the target variable in terms of input attributes. The flowchart of GEP modelling is shown in Figure 5. The setting parameters such as number of chromosomes, genes, and head size were varied to find the optimized hyperparameters. Finally, 30 chromosomes, 3 genes, and 10 head sizes resulted in the best model.

## 3. Results and Discussions

### 3.1. Experimental Results

**Figure 6:**
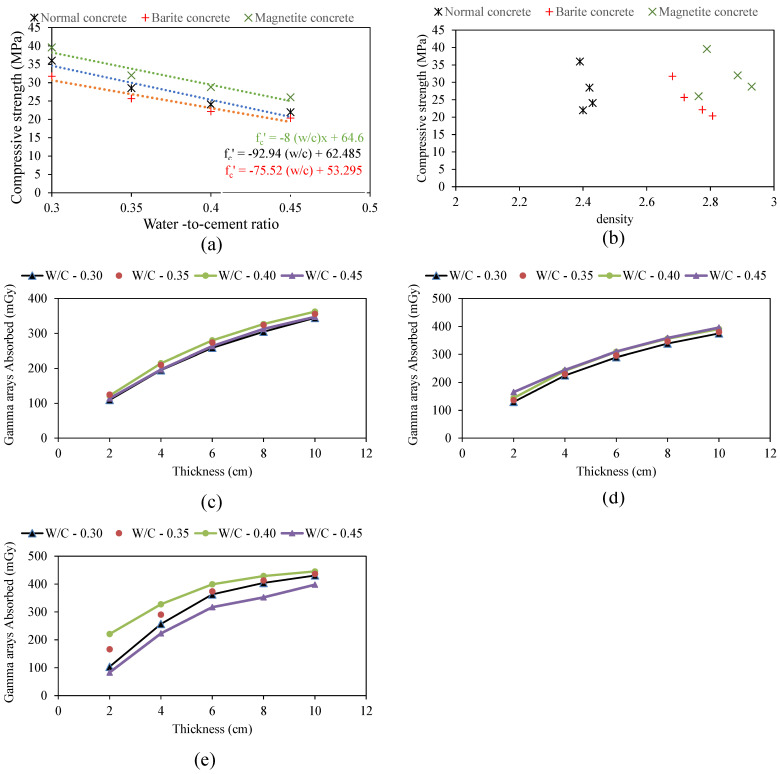
Results obtained from experimental study: (**a**,**b**) compressive strength versus water to cement ratio and density, (**c**) thickness versus gamma rays absorbed for normal concrete, (**d**) thickness versus gamma rays absorbed for barite concrete, and (**e**) thickness versus gamma rays absorbed for magnetite concrete.

**Figure 7:**
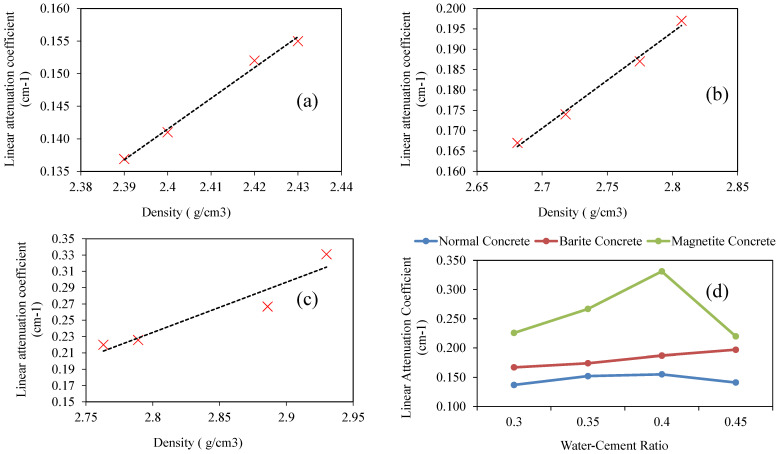
Variation of linear attenuation coefficient with density: (**a**) normal concrete, (**b**) barite concrete, (**c**) magnetite concrete, and (**d**) linear attenuation versus water to cement ratio.

The results of compressive strength for the three types of concrete investigated in this study are shown in Figure 6a. Overall, the addition of barite has reduced the compressive strength, whereas the addition of magnetite has increased the compressive strength. The variation with *w*/*c* ratio is evident, reflecting a linear reduction of compressive strength with an increase in *w*/*c* ratio from 0.30 to 0.45. The variation in strength with change in *w*/*c* ratio is consistent with a number of previous studies [65,66]. The variation with the density is no more pronounced as depicted in Figure 6b for the three different types of concrete. Figure 6c–e shows the variation of the dose absorbed by the sample. It is evident that the thickness of concrete is a vital parameter in designing the shielding capacity of concrete. Normally, the radiation shielding capacity is denoted by the linear attenuation coefficient. The linear attenuation coefficient was computed for concrete specimens with different *w*/*c* ratios and is presented in Figure 7. It is evident that a linear relation exists between the thickness of the concrete sample and the linear attenuation coefficient. Linear attenuation was observed to have increased with a rise in *w*/*c* ratio up to 0.40, followed by a decrease. The maximum value of the attenuation coefficient is obtained at *w*/*c* equal to 0.40. Figure 7 presents the relation between density and linear attenuation coefficient. There is a linear relationship between the density of concrete and the linear attenuation coefficient. The maximum value of the attenuation coefficient at a *w*/*c* ratio of 0.40 may be attributed to higher concrete density. So, it can be inferred from the test results that gamma rays can be attenuated either by using lighter materials with an increased thickness or heavier materials with optimum thickness. Normal concrete can be used as a useful material for gamma-ray shielding if it is used at a *w*/*c* ratio at which concrete has maximum density. The results are in accordance to the literature related to different materials used for shielding against radiation; specifically, studies that used high density constituent in concrete, significantly increased the attenuation coefficient [25,26,27,28,32,33,37,38].

### 3.2. Performance of the Models

**Figure 8:**
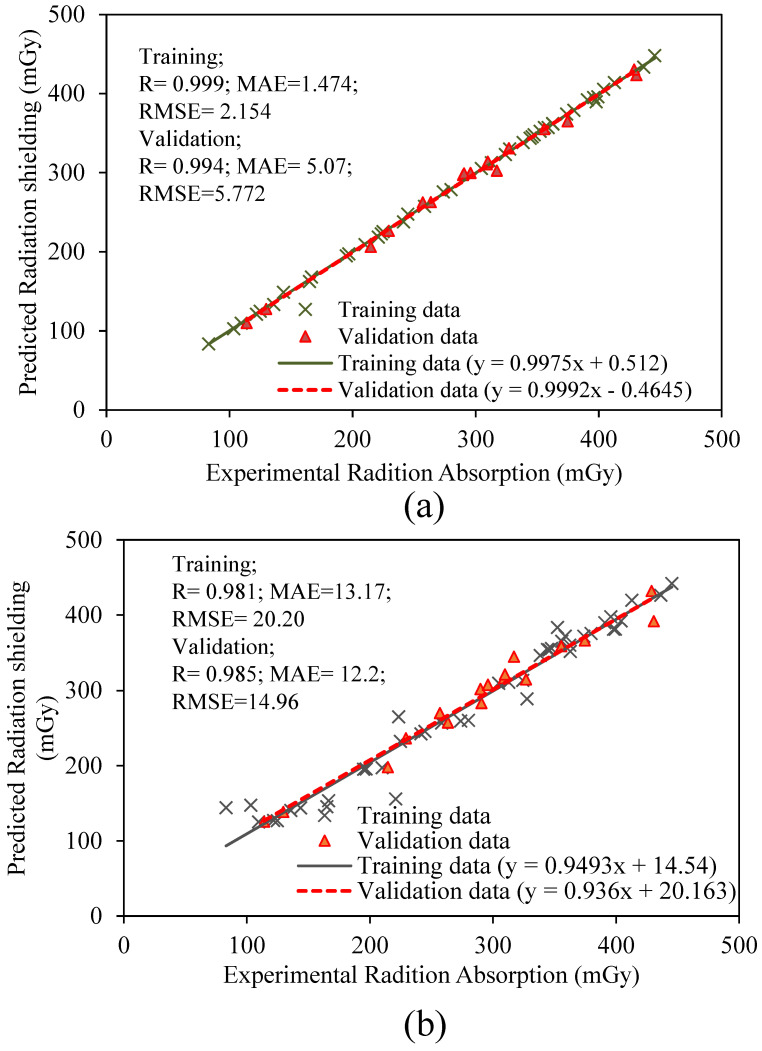
Comparison of experimental versus predicted results in the form of regression slopes and statistical indices for the: (**a**) ANN model, and (**b**) GEP model.

The developed ANN and GEP models were evaluated using statistical indices, i.e., the values of R, MAE, and RMSE, provided in Figure 8, in accordance with [60,61,62,64,67,68,69,70,71,72,73,74]. The R values of 0.999 and 0.994 were observed for training and validation data, respectively, for the ANN model (Figure 8a), whereas, for the GEP model, these values were noticed as 0.981 and 0.985 (Figure 8b). The MAE value of 1.474 mGy and 13.17 mGy were obtained for the training data while MAE values of 2.154 mGy and 12.2 mGy were observed for validation data of the ANN and GEP models, respectively. The results for RMSE values are also presented in Figure 8. It is evident that the values of R for the ANN and GEP models are very close to each other; however, the error indices in the case of the ANN model show a more robust performance of the model. The error indices obtained from the GEP model are also acceptable; however, the error indices are less accurate as compared to the ANN model.

While investigating the performance in terms of regression slope, experimental values of the radiation absorbed were plotted on the *x*-axis, and the predicted values were plotted on the *y*-axis. Many researchers in previous studies used the slope of the regression line as a statistical evaluation procedure for studying the performance of AI models [62,64,71]. Previously, the researchers argued that the values of regression slope of more than 0.80 represent the close agreement of experimental and predicted values [71,73,74]. It was found that the ANN model interpreted slopes equaling 0.9975 and 0.9992 for the training and validation sets. Similarly, the GEP model manifested slopes equaling 0.9493 and 0.936 for the training and validation data. These observations also prove that the slopes are closer to the ideal slope (equal to 1). Moreover, it also shows that the ANN model resulted in relatively accurate prediction compared to the GEP model. Khan et al. [71] used the ANN model for investigating the compressive strength of polyethylene terephthalate-incorporated cementitious grouts and observed a slope value of 1.01 for training and 0.90 for testing data. This result was noted while evaluating the ANN non-linear abilities for compressive strength.

**Figure 9:**
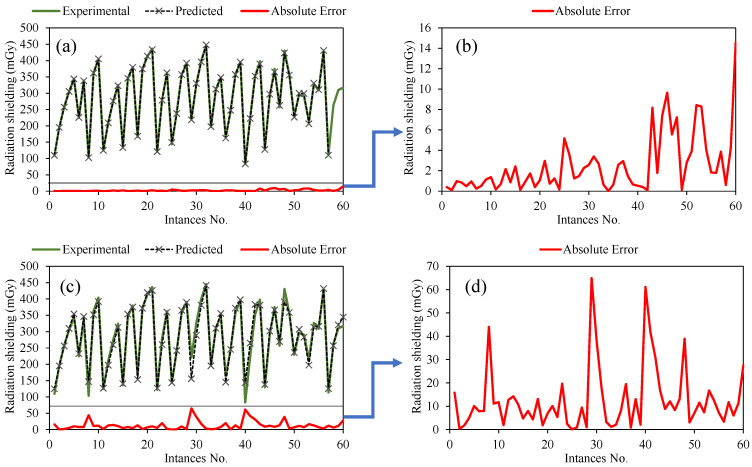
Error analysis of the developed models: (**a**) tracing of experimental by predictions for the ANN model, (**b**) absolute error from the ANN model, (**c**) tracing of experimental by predictions for the GEP model, and (**d**) absolute error from the GEP model.

The model statistical assessment was further enhanced through the error analysis and the tracing of experimental results by the predictions made from the ANN and GEP models. The results relating to this analysis are presented in Figure 9. Both the models traced the experimental results very closely (Figure 9a,c); however, in the case of the ANN model, the proximity is very close compared to the GEP model. This result is evident from the error analysis shown in Figure 9b,d, which shows that the error ranged from 0 to 14.56 mGy in the ANN model, whereas, for the GEP model, it ranged from 0 to 64.6 mGy. It can be derived from error analysis that most of the points converged around zero error, with a maximum deviation of 14.5 mGy and 64.6 mGy in the ANN and GEP model, respectively.

It is obvious from the above analysis that ANN performs better in terms of accuracy; however, the GEP model has interpreted acceptable results. The beauty of the GEP model is lying in the fact that it furnishes a simple mathematical equation, which can be used for predicting a new data set, without using a computer program. The disadvantage of the ANN model is its black-box nature. You must retrain the model with the data used originally in order to predict the new data. Equations are given as follows (Equations (2)–(5)), obtained from the GEP model which can be used for predicting the radiation dose absorbed for a given sample of concrete whose input parameters are known.

(2) y=a+b+c

(3) $$a=((\overset{´}{f_{c}}+5.94+D{)}^{2}+\left(\left(w/c{)}^{2}\times \left(D-T\right)-12.94\right)\right)$$

(4) $$b=((1.49^{2}\times {\overset{´}{f_{c})}}^{2}+((-11.02\times (w/c))+8.75D-w/c))$$

(5) $$c=\left((\left({\overset{´}{f_{c}}}^{2}-D\right)\times (D-1.52\right)\times (w/c)\times 2.044)-8.56)$$

### 3.3. Sensitivity and Parametric Analysis

**Figure 10:**
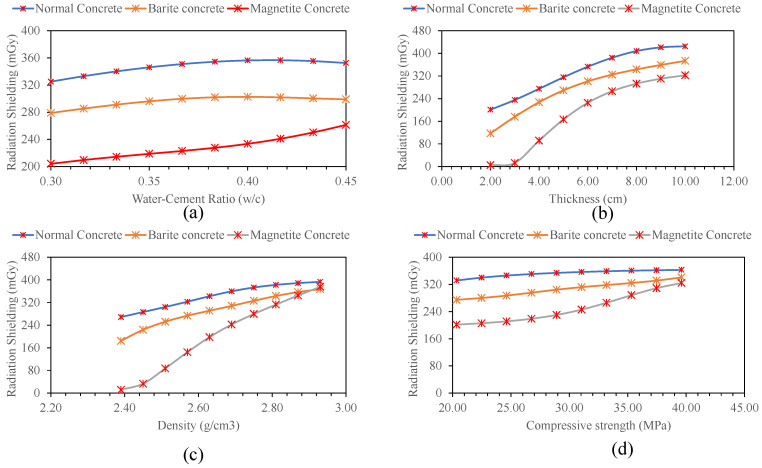
Parametric analysis of ANN model for predicting radiation shielding capacity with: (**a**) water-cement ratio, (**b**) thickness, (**c**) density of concrete, and (**d**) compressive strength of concrete.

Sensitivity and parametric analysis of the developed model is the evaluation of the trained model on the entire new dataset. The sensitivity analysis shows which input variable is more important in furnishing the magnitude of the target variable, and the parametric analysis depicts the trend in contributing towards the output variable. For this purpose, a simulated dataset was created such that one input variable was varied between its extreme values, whereas the other variables were kept constant at their average values. The simulated dataset was created for the three types of concrete investigated in this study. The simulated dataset was tested on the trained ANN model owing to the superior performance of the model. The results are shown in Figure 10 for normal concrete (type-1), barite concrete (type-II), and magnetite concrete (type-III), respectively.

The parametric study revealed that maximum radiation is absorbed at *w*/*c* ratio of 0.40–0.42 for normal and barite containing concrete, and 0.45 for magnetite-containing concrete. The increase in thickness and density also improved the radiation shielding depicting polynomial variation. Maximum radiation shielding was observed for the maximum compressive strength investigated in this study. It is highly recommended to consider these input parameters and their effect on radiation shielding structures intended to resist radiation especially atomic power plants.

**Figure 11:**
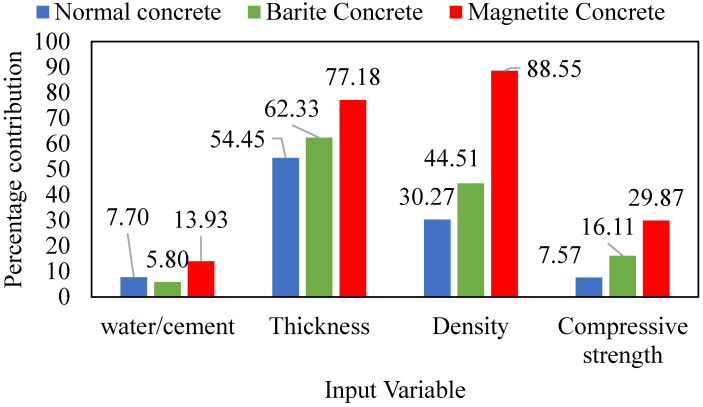
Sensitivity analysis of ANN model.

The sensitivity analysis (Figure 11) showed that the thickness of concrete is the most influential variable in estimating the radiation shielding capacity of concrete in the case of normal and barite containing concrete, followed by the density of the concrete. In the case of magnetite concrete, density played significant role in contributing radiation shielding of concrete. The compressive strength and *w*/*c* ratio were observed showing least contribution in radiation shielding.

## 4. Conclusions

This study examined the shielding capability and mechanical properties of concrete at variable water-to-cement ratio, thickness, density, and compressive strength. For this purpose, three different types of concrete, i.e., normal concrete, barite, and magnetite-containing concrete, were cast and tested for compressive strength and radiation shielding. Moreover, ANN and GEP models were developed to predict radiation shielding of concrete. The following results were drawn from this study:

- The density of material and the thickness of concrete samples are the two important factors that attenuate the quantity of gamma radiation. Increasing the thickness and density of concrete improves its radiation shielding ability. Normal concrete is the most commonly used material in construction. Therefore, it is recommended to use such a *w*/*c* ratio in order to achieve a higher density without affecting strength properties. For normal and barite-containing concrete, the optimum water/cement ratio was observed as 0.40–0.42. Using an optimal *w*/*c* ratio would decrease the wall thickness required for radiation shielding in therapy bunkers and atomic reactors, indirectly saving cost. Increasing the *w*/*c* ratio was found to reduce the compressive strength.
- The AI models developed in this study showed close agreement between experimental and predicted results; however, the ANN model developed for predicting radiation shielding manifests outperformed the GEP model. The simple mathematical relationship produced from the GEP model signifies its importance because it can be used in the future to predict radiation shielding of new data without using any computer program.
- The results obtained from the parametric analysis showed agreement with the experimental results. The thickness and density of concrete were found to be the most influential parameters in determining the shielding ability of concrete.

## Figures and Tables

**Table 1:** Physical properties of cement, coarse aggregate, and fine aggregate.

	Ingredient	Physical properties				
		Specific Gravity	Bulk Density	Absorption Capacity	Max. Aggregate Size	Fineness Modulus
Cement		3.15	-	-	-	-
Coarse Aggregates		2.65	1602 kg/m^3^	1.30%	25.44 mm	-
Fine Aggregates		2.38	-	1.88%	-	2.34
Barite		3.10	-	3.05%	-	2.31
Magnetite		3.20	-	0.91%	-	3.20

**Table 2:** Chemical composition of Portland cement (XRD result).

Constituents	Mass Percentage (%)
CaO	77.2
SiO_2_	15.8
Al_2_O_3_	5.82
Fe_2_O_3_	1.06
ZnO	0.07
MnO + TiO_2_ + K_2_O	0.05

**Table 3:** Mix properties.

S. No.	Properties	Value
1	Slump range	25–50 mm
2	Maximum size of coarse aggregate	25 mm down
3	Bulk density of coarse aggregate	591.1 kg/m^3^
4	Specific gravity of coarse aggregate	2.65
5	Absorption capacity of coarse aggregate	1.3%
6	Specific gravity of sand	2.38
7	Fineness modulus of sand	2.34
8	Absorption capacity of sand	2%
9	Specific gravity of cement	3.15

**Table 4:** Mix proportioning of concrete.

Water/Cement (*w*/*c*)	Weight of Concrete Ingredients (Kg/m^3^)						Chemical Admixture (%)
	Water	Cement	Fine Aggregates			Coarse Aggregates	
			Sand	Barite	Magnatite		
0.30	178	593	491	639.5	660.2	1117	1.5
0.35	208	593	462	601.8	621	1117	1
0.40	237	593	432	562.7	580.8	1117	0.8
0.45	267	593	402	523.6	540.5	1117	0.5

**Table 5:** Concrete workability test results.

*w*/*c*	Slump Type	Slump Values (mm)
0.30	True	36
0.35	True	63
0.40	True	74
0.45	True	89

**Table 6:** Training dataset for model development.

Input Variables					Output Variable
Concrete Type	*w*/*c*	Thickness (cm)	Density (g/cm^3^)	Compressive Strength (MPa)	Gamma Rays Absorption (mGy)
1	0.30	2	2.39	35.99	109.47
1	0.30	4	2.39	35.99	194.77
1	0.30	6	2.39	35.99	258.37
1	0.30	8	2.39	35.99	304.57
1	0.30	10	2.39	35.99	344.27
2	0.30	4	2.68	31.76	224.67
2	0.30	8	2.68	31.76	338.47
3	0.30	2	2.79	39.60	103.24
3	0.30	6	2.79	39.60	362.78
3	0.30	8	2.79	39.60	403.99
1	0.35	2	2.42	28.50	124.77
1	0.35	4	2.42	28.50	209.97
1	0.35	6	2.42	28.50	273.67
1	0.35	8	2.42	28.50	324.077
2	0.35	2	2.72	25.65	135.77
2	0.35	8	2.72	25.65	345.77
2	0.35	10	2.72	25.65	379.77
3	0.35	2	2.89	32.00	166.46
3	0.35	6	2.89	32.00	373.82
3	0.35	8	2.89	32.00	412.79
3	0.35	10	2.89	32.00	436.400
1	0.40	2	2.43	24.06	121.77
1	0.40	6	2.43	24.06	279.97
1	0.40	10	2.43	24.06	362.47
2	0.40	2	2.78	22.15	143.67
2	0.40	4	2.78	22.15	241.37
2	0.40	8	2.78	22.15	355.87
2	0.40	10	2.78	22.15	390.77
3	0.40	2	2.93	28.80	220.83
3	0.40	4	2.93	28.80	327.57
3	0.40	6	2.93	28.80	399.34
3	0.40	10	2.93	28.80	445.35
1	0.45	4	2.40	21.98	196.87
1	0.45	8	2.40	21.98	312.77
1	0.45	10	2.40	21.98	347.47
2	0.45	2	2.81	20.34	164.97
2	0.45	4	2.81	20.34	244.77
2	0.45	8	2.81	20.34	358.77
2	0.45	10	2.81	20.34	395.77
3	0.45	2	2.76	26.00	83.04
3	0.45	4	2.76	26.00	223.24
3	0.45	8	2.76	26.00	352.41
3	0.45	10	2.76	26.00	397.88
2	0.30	2	2.68	31.76	129.67
2	0.30	6	2.68	31.76	289.77
2	0.30	10	2.68	31.76	374.67

**Table 7:** Validation dataset used in artificial neural network (ANN) modelling.

Concrete type	*w*/*c*	Thickness (cm)	Density (g/cm^3^)	Compressive Strength (MPa)	Gamma Rays Absorption (mGy)
3	0.30	4	2.79	39.60	256.89
3	0.30	10	2.79	39.60	430.69
1	0.35	10	2.42	28.50	355.57
2	0.35	4	2.72	25.65	229.27
2	0.35	6	2.72	25.65	295.87
3	0.35	4	2.89	32.00	290.61
1	0.40	4	2.43	24.06	214.67
1	0.40	8	2.43	24.06	326.87
2	0.40	6	2.78	22.15	308.97
3	0.40	8	2.93	28.80	428.79
1	0.45	2	2.40	21.98	113.87
1	0.45	6	2.40	21.98	263.40
2	0.45	6	2.81	20.34	309.77
3	0.45	6	2.76	26.00	317.10

## Data Availability

The data used in this research has been properly cited and reported in the main text.
